# Pineal parenchymal tumour of intermediate differentiation: a rare differential diagnosis of pineal region tumours

**DOI:** 10.1259/bjrcr.20150371

**Published:** 2016-11-02

**Authors:** Daniel J Yoon, James Park, Lhara M Lezama, Gordon D Heller

**Affiliations:** ^1^Department of Radiology, Mount Sinai West Hospital Center of the Icahn School of Medicine, New York, NY, USA; ^2^Department of Pathology, Mount Sinai West Hospital Center of the Icahn School of Medicine, New York, NY, USA

## Abstract

Pineal parenchymal tumours of intermediate differentiation are a rare type of pineal parenchymal tumours. As indicated by their name, these tumours fall between pineoblastoma (a malignant pineal parenchymal tumour) and pineocytoma (a benign pineal parenchymal tumour). In this article, we present a case of pineal parenchymal tumour of intermediate differentiation that was successfully treated by resection *via* the supracerebellar approach. We also discuss the differential consideration based on epidemiological, pathological and radiological findings.

## Clinical presentation

**Figure 1.**
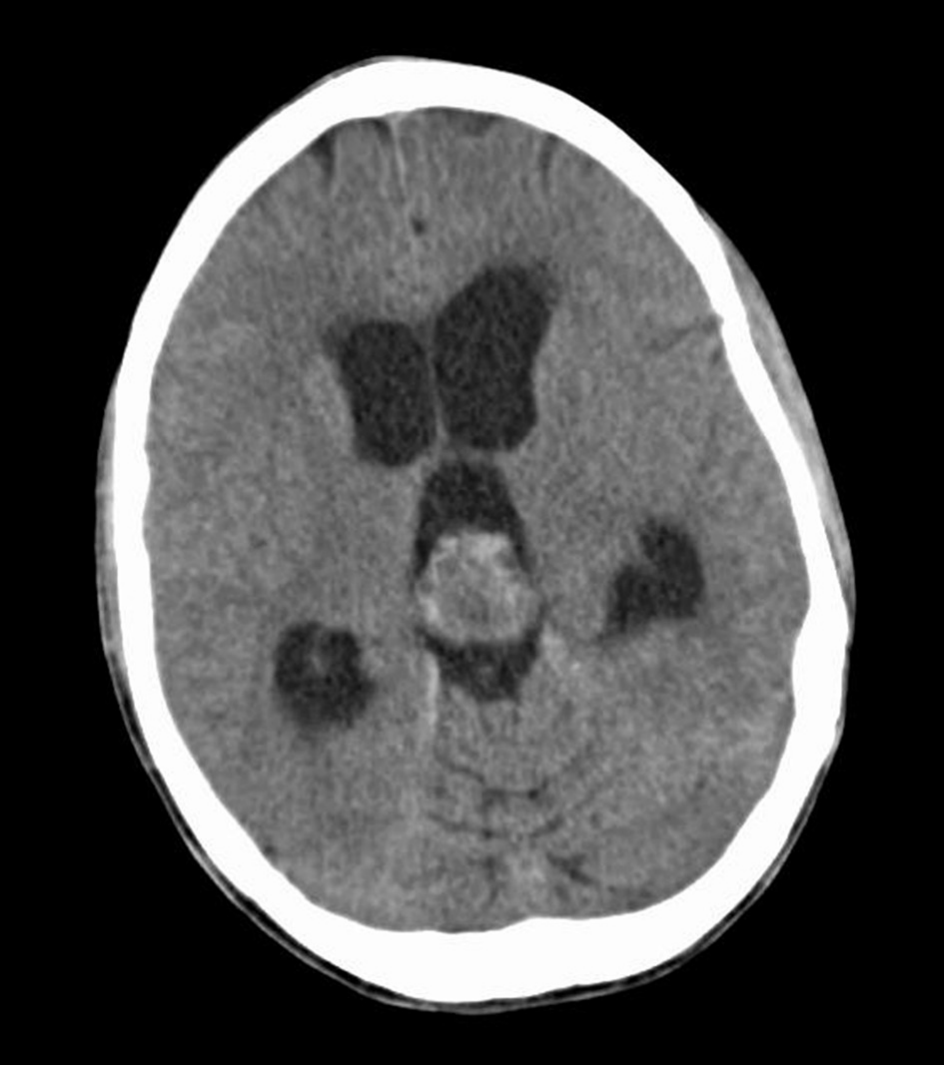
A non-contrast CT scan of the head demonstrates a heterogeneous mass in the pineal gland, resulting in obstructive hydrocephalus of the third and lateral ventricles.

**Figure 2.**
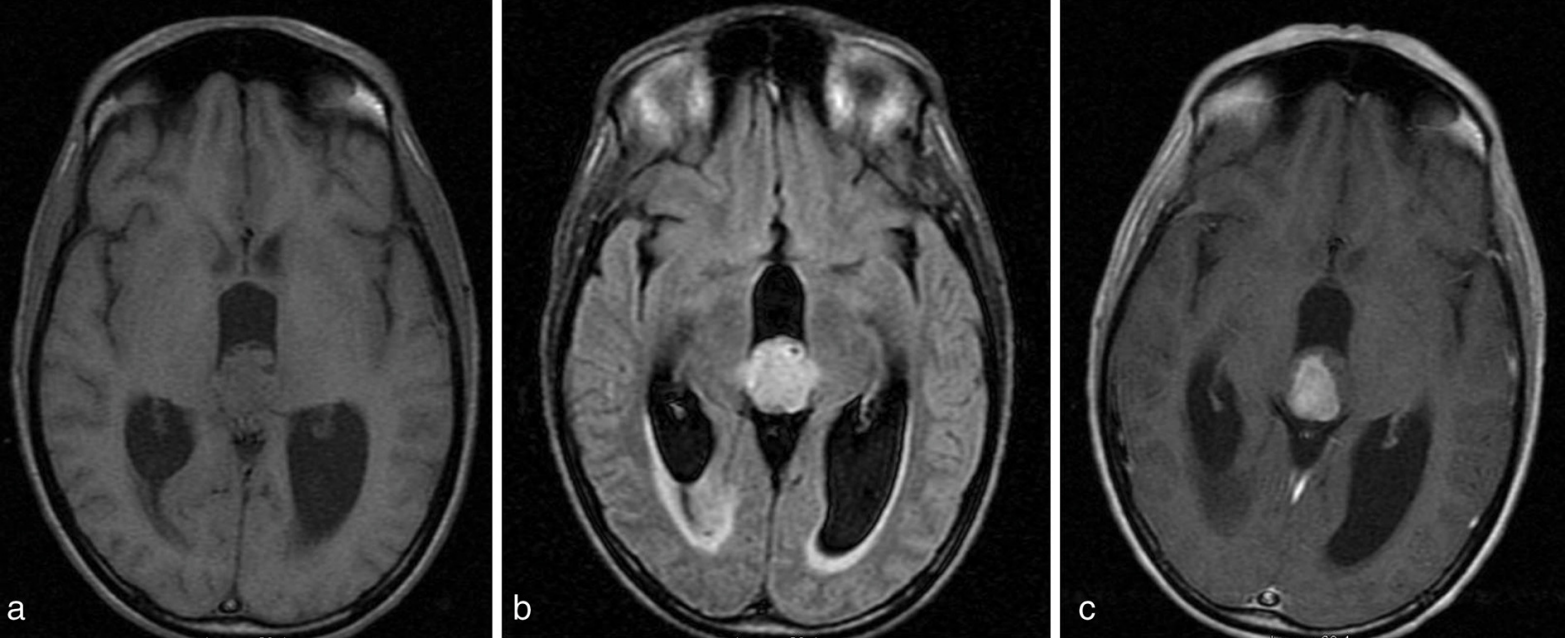
MRI of the brain with and without contrast demonstrates a mildly lobulated, well-circumscribed, *T*_1_ hypo/isointense (a), *T*_2_ hyperintense (b) soft tissue mass with enhancement (c) in the pineal region. A small non-enhancing component is seen in the anterior aspect of the tumour.

**Figure 3.**
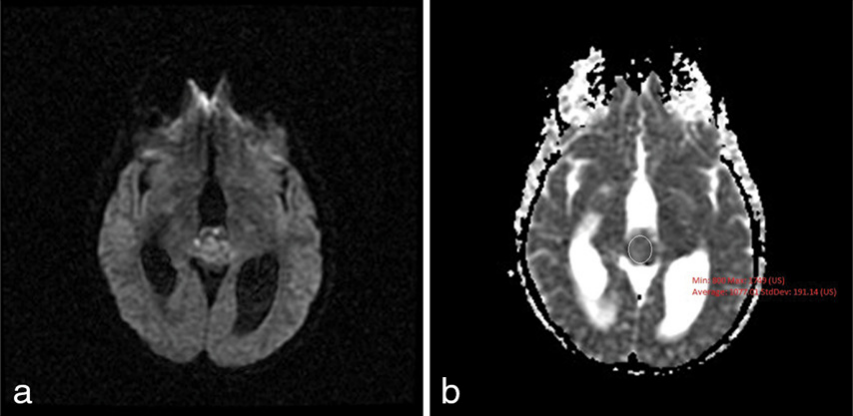
Diffusion weighted images (a) and apparent diffusion coefficient map (b) show an apparent diffusion coefficient value of 1077.01 × 10^−6^ mm^2^ s^−1^ ± 191.14 mm^2 ^s^−1^.

**Figure 4.**
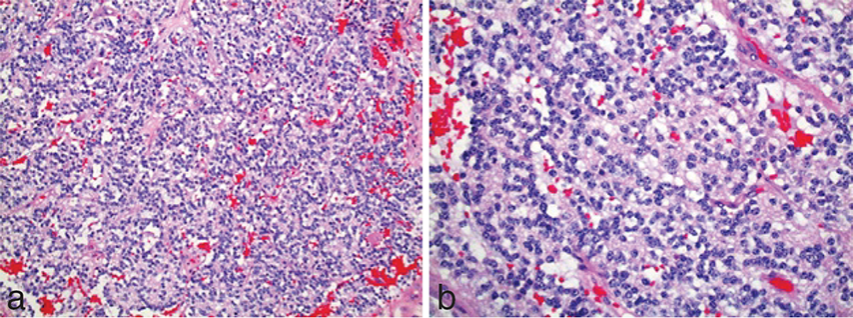
Low power magnification shows a moderately cellular tumour forming sheets and pseudo-rosette patterns with absence of well-formed rosettes (a). High power magnification shows relatively uniform cells with weakly eosinophilic cytoplasm, round nuclei and granular chromatin (b).

A 25-year-old female with history of chronic headaches and Kawasaki’s disease presented to the emergency department with lethargy, blurry vision, confusion and headache. Initial laboratory results showed mild hyponatraemia (127 mmol l^−1^). The remaining metabolic panel and complete blood count results were normal. Non-enhanced CT imaging showed an approximately 2.2 cm heterogeneous mass without calcification in the pineal gland region (Figure 1). Obstructive hydrocephalus with transependymal cerebrospinal fluid resorption was noted and an extraventricular drain was placed emergently. MRI of the brain was subsequently performed (1.5 T GE magnetic resonance scanner, Chicago, IL). MRI demonstrated a lobulated mass with *T*_1_ signal characteristics isointense to brain parenchyma. Fluid attenuation inversion recovery images demonstrated the mass to be hyperintense (Figure 2). The tumour demonstrated enhancement following gadolinium administration, except for a small non-enhancing component in the anterior aspect of the tumour. A continuous elliptical region of interest was placed manually on the apparent diffusion coefficient (ADC) map of the tumour. The ADC value of the tumour was 1077.01 × 10^−6^ mm^2^ s^−1 ^ ± 191.14 mm^2 ^s^−1^ (Figure 3), which is higher than the normal brain tissue value. There was compression of the tectal plate and obstructive hydrocephalus with transependymal cerebrospinal fluid flow (Figure 2). Perfusion and spectroscopy images were not obtained, as these were not part of the routine brain MRI protocol. MRI of the spine was also performed and showed no evidence of extracranial tumour involvement. The patient underwent a suboccipital craniectomy *via* a supracerebellar approach in order to resect the pineal mass. Histological examination revealed a moderately cellular tumour forming sheets and pseudo-rosette patterns, comprising relatively uniform cells with weakly eosinophilic cytoplasm, round nuclei and granular chromatin. Well-formed rosettes were absent. The mitotic index was low—less than 6 mitosis per 10 higher power fields. Immunohistochemical staining showed diffuse positivity for neuron-specific enolase and focal positivity for neurofilament and synaptophysin (Figure 4). Given these findings, a diagnosis of pineal parenchymal tumours of intermediate differentiation (PPTID) was made. The patient was symptom-free at 1-year follow-up, and follow-up MRI of the brain, with and without contrast, showed no residual enhancing mass and resolution of the obstructive hydrocephalus.

## Discussion

### Introduction

Pineal region tumours are uncommon and account for less than 1% of all intracranial tumours.^1^ Of these tumours, the majority are germ cell in origin and include germinomas, embryonal cell tumours and choriocarcinomas.^2^ Pineal parenchymal tumours arise from pineocytes (or their precursors) and are the second most common subgroup. Before 2007, only two subtypes of pineal parenchymal tumours were recognized by the World Health Organization (WHO): pineocytomas (WHO Grade I) and pineoblastomas (WHO Grade IV). In 2007, PPTID was established as a distinct entity to categorize a group of tumours that were between pineoblastomas and pineocytomas in histological grade.^3^ Although pineoblastomas and pineocytomas comprise the majority of pineal parenchymal tumours, PPTID have reported rates between 10−20%.^4^ Prior to the official WHO classification, tumours that fell in the spectrum between pineoblastomas and pineocytomas were described by various terms such as “atypical pineocytomas,” “malignant pineocytomas” or “mixed pineocytoma–pineoblastomas”.^5^ It is now thought that many of these neoplasms were likely PPTID.

The clinical presentation of a PPTID is similar to that of other pineal region masses. Diplopia and headache are the most common symptoms. Parinaud’s syndrome (vertical gaze disturbance due to compression of the tectal plate) is another common finding. If large enough, PPTID can cause hydrocephalus, leading to associated symptoms of elevated intracranial pressure such as ataxia.^6^ PPTID have a broader patient age spectrum. In one series of 11 cases, the age range was 4–75 years, with a mean of 23 years. There appears to be a slight female preponderance, as seen in our case.^4^

Histologically, PPTID appear as diffuse sheets of small uniform cells and are characterized by moderate-to-high cellularity, mild-to-moderate nuclear atypia and low-to-moderate mitotic activity. Absence of pineocytomatous rosettes should be noted.^3^ On immunohistochemical staining, these neoplasms are strongly positive for synaptophysin and neuron-specific enolase with variable positivity for neurofilament protein, chromogranin A, retinal S-antigen, S-100 protein and B-tubulin.^7^ Owing to only a limited number of reported cases, histological grading remains controversial, although most agree that PPTID are WHO Grade II or III. Jouvet et al^7^ proposed a grading system where tumours with < 6 mitoses and positive immunolabelling for neurofilaments were categorized as Grade II, whereas tumours with > 6 mitoses without immunolabelling for neurofilaments were categorized as Grade III neoplasms.

### Differential diagnosis

The differential diagnosis of PPTID includes other pineal parenchymal tumours (pineocytomas and pineoblastomas), germ cell tumours and papillary tumours of the pineal region (Table 1).

**Table 1.** Demographics and radiological findings of pineal tumours

Tumour types		Demographics	Typical imaging findings
Pineal parenchymal tumours	Pineocytomas	Young adults, after second decade of life	Well-circumscribed, homogeneously enhancing mass. Tends to be solid; however, cystic degeneration can occur
	Pineal parenchymal tumours of intermediate differentiation	Broad age spectrum, with mean age in the 20s	More locally invasive and heterogeneous than pineocytomas. Heterogeneous enhancement. CSF seeding can occur
	Pineoblastomas	Mostly affects the paediatric population	Large, poorly defined mass. Peripheral calcifications in “exploded” pattern. Prone to CSF seeding
Germ cell tumours	Germinomas	Mean age in the second decade (10−19 years)	Soft tissue density mass, isodense to gray matter, with homogeneous enhancement. More central calcification “engulfed” pattern
	Teratomas	Children, young adults	Heterogeneous mass containing various tissue types, including fat and calcium
Papillary tumours		Broad age spectrum, with mean age in the 30s	Mildly enhancing *T*_1_ hyperintense lesion. May contain cystic components

CSF, cerebrospinal fluid.

### Pineal parenchymal tumours

Pineocytomas tend to affect young adults who are beyond their second decade of life.^8^ Radiologically, pineocytomas are well-circumscribed, slow-growing tumours with homogeneous enhancement. Although pineocytomas tend to be solid lesions, cystic changes may occur.^2^ Pineoblastomas are more likely to occur in the paediatric population than in adults.^1^ There is no gender preponderance.^8^ Pineoblastomas are highly malignant and tend to be larger and poorly defined tumours. Characteristically, calcification changes of pineal parenchymal tumours tend to occur in the periphery and are described as “exploded” calcifications.^2,4^

Several recent studies have attempted to describe the imaging characteristics of PPTID, although these are not firmly established owing to their recent recognition as a distinct pineal neoplasm. Generally, PPTID are lobulated, vascular pineal region masses that can extend into adjacent structures such as the ventricles or thalami. Owing to high cellularity, PPTID are usually hyperdense on CT scans and can demonstrate peripheral exploded calcifications. On MRI, these tumours are heterogeneously hypointense on *T*_1_ weighted and heterogeneously hyperintense on *T*_2_ weighted images. Cystic areas can be seen within the tumour as well. Heterogeneous enhancement is typical. Hydrocephalus is often seen owing to mass effect on the tectum. One case series found that 80% of PPTID had local invasion.^4^ Rarer complications include intracranial dissemination and cerebrospinal fluid spread to the spine.^1^ Overall, as in the presenting case and description by Komakula et al,^4^ PPTID are likely to be larger, more heterogeneous and more likely to be locally invasive than pineocytomas, and appear to be less likely to result in subarachnoid and spinal seeding than pineoblastomas.^4^

As mentioned previously, germ cell tumours are the most common type of tumour of the pineal region. Incidence of germinomas peak during the second decade of life, and there is a male preponderance.^8^ In non-contrast CT examinations, germinomas tend to be isodense to the gray matter, with homogeneous enhancement after i.v. contrast administration.^2^ MRI demonstrates a mass that has *T*_1_ and *T*_2_ signal intensity similar to the surrounding brain parenchyma. They tend to surround or engulf the pineal gland and cause calcification changes of the gland within the tumour itself.^2^ This “engulfed” pattern of calcification has been shown to be useful in differentiating germ cell tumours from pineal parenchymal tumours.^8^ In addition, germ cell tumours tend to have a higher ADC value than the pineal parenchymal tumours, likely secondary to lesser tumour cellularity and lesser nuclear/cytoplasmic ratio. Dumrongpisutikul et al^9^ used a threshold value of less than 1250.00 mm^2^ s^−1^ to differentiate the pineal parenchymal tumours from germ cell tumours.^9^ Our case’s ADC value of 1077.01 × 10^−6^ mm^2^ s^−1 ^ ± 191.14 mm^2^ s^−1^ is consistent with the said proposal.

Teratomas are the second most common pineal tumour. They have a male preponderance and affect children and young adults.^8^ Although teratomas have a heterogeneous CT and MRI appearance, the presence of fat is suggestive of teratoma.^2^ Choriocarcinomas are hypervascular and commonly haemorrhagic, which may result in susceptibility artefact in gradient echo sequences.^8^ Other non-germinomatous germ cell tumours, such as yolk sac tumours and embryonal carcinomas, are rarer and lack specific imaging characteristics.^2^

Papillary tumours of the pineal region are extremely rare tumours, with less than 100 cases reported in the literature. The largest retrospective study of this disease entity demonstrated the mean age of the patients to be 31.5 years (range 5–66 years), with a slight female preponderance.^10^ Radiologically, papillary tumours have been described as mildly enhancing *T*_1_ hyperintense lesions with cystic components.^2^

## Treatment

When presenting with symptoms of hydrocephalus, decompression of the ventricular system assumes the highest priority. Although optimal treatment has yet to be established, most cases with locally limited disease are treated with surgical resection.^11^ The addition of adjuvant chemotherapy and craniospinal radiation is typically reserved for tumours with local invasion and/or disseminated disease. Radiotherapy is performed with fractionated external beam radiation, Gamma Knife radiosurgery or brachytherapy.^11^ This is in contrast to pineocytoma, which is optimally treated with surgical resection alone, and to pineoblastoma, which is frequently treated with radical surgery in conjunction with neoadjuvant chemotherapy and/or radiation.^12^

## Learning points

1. PPTID are a rare type of pineal parenchymal tumours.
2. Owing to their relatively recent recognition, imaging characteristics of PPTID are not firmly established. However, PPTID are likely to be larger, more heterogeneous and more likely to be locally invasive than pineocytomas and appear to be less likely to result in subarachnoid and spinal seeding than pineoblastoma.
3. Radiologists need to be aware of PPTID as a part of the differential diagnosis of pineal region tumour, as PPTID may follow a different treatment plan from other pineal parenchymal tumours.

## Consent

Written informed consent was obtained from the patient for publication of this case report, including accompanying images
